# The safety and efficacy of carbon nanoparticle suspension injection versus indocyanine green tracer-guided lymph node dissection during radical gastrectomy (FUTURE-01): A single-center randomized controlled trial protocol

**DOI:** 10.3389/fonc.2022.1044854

**Published:** 2023-01-06

**Authors:** Yuan Tian, Yue Pang, Peigang Yang, Honghai Guo, Yang Liu, Ze Zhang, Pingan Ding, Tao Zheng, Yong Li, Liqiao Fan, Zhidong Zhang, Xuefeng Zhao, Bibo Tan, Dong Wang, Qun Zhao

**Affiliations:** Third Surgery Department, The Fourth Hospital of Hebei Medical University, Shijiazhuang, Hebei, China

**Keywords:** clinical trial, design, gastric cancer, indocyanine green, lymph node, carbon nanoparticle suspension injection, gastrectomy, protocol

## Abstract

**Background:**

The use of lymph node (LN) tracers can help obtain a complete dissection of the lymph nodes and increase the detection rate of LNs and metastatic LNs. Carbon nanoparticle suspension injection (CNSI) and indocyanine green (ICG) have been widely used in radical gastrectomy in recent years. Nevertheless, the comparison of their clinical effects has not been studied.

**Method/design:**

The FUTURE-01 trial will be the first randomized, open-label, single-center trial to compare CNSI and ICG. The study started in 2021 and enrolled 96 patients according to a prior sample size calculation. The primary outcome is the number of LNs retrieved. The secondary outcomes are LN staining rate, LN metastasis rate, stained LN metastasis rate, perioperative recovery and survival.

**Conclusion:**

By comparing the safety and efficacy of CNSI and ICG tracer-guided LN dissection in patients with gastric cancer, we can determine the most appropriate LN tracer at present. With the help of LN tracers, the operation is simplified, and the prognosis of these patients is improved. Our study is a prospective exploration of the safety, efficacy, and prognosis of CNSI and ICG.

**Clinical trial registration:**

https://clinicaltrials.gov/ct2/show/NCT05229874?cond=NCT05229874&draw=2&rank=1, identifier NCT05229874.

## 1 Introduction

Gastric cancer (GC) is one of the most common malignancies worldwide (1). Despite the global downward trends in the GC incidence and mortality rates, GC remains one of the most common causes of death by cancer worldwide and ranks second (2).

For GC, complete resection with standardized LN dissection (D2) is important (3). Although LN dissection is deemed the crucial step in radical gastrectomy, there is no consensus regarding the number of LNs detected worldwide (4–6). The quality of LN dissection should be evaluated by the number of LNs detected, and more detection of LNs can reduce the N staging bias and prolong the GC patient prognosis (7–9).

D2 LN dissection is complex and tricky, and it is performed by removing perivascular fat and the LNs containing adipose baring fat tissue. In the process of lymphadenectomy in gastrectomy, one of the common and severe intraoperative complications is vessel injury. With the aim of easing the difficulty of LN dissection, an increasing number of surgeons are trying to use LN tracers to make the affected LNs distinct. Initially, surgeons used methylene blueand tattoo ink as LN tracers (10). Because of the difficulty of time limitations and low resolution, researchers have gradually abandoned the use of methylene blue.

Currently, two kinds of novel LN tracers have been monitored by endoscopy experts and surgeons. One is carbon nanoparticle suspension injection (CNSI), and the other is indocyanine green (ICG). In 2004, the CNSI (Carnaline, Chongqing Lummy Pharmaceutical Co., Ltd) made in China was launched, which reduced the price of CNSI and the cost medical expenditure and promoted the development of LN tracers. This product is a stable suspension of carbon nanoparticles 150 nm in diameter, which are smaller than the lymphatic capillary endothelial cell gap (120-500 nm) and larger than the capillary endothelial cell gap (30-50 nm) (11). Therefore, following their injection into the submucosa of the gastric wall around the tumor, the carbon nanoparticles rapidly captured by macrophages cannot enter into the blood vessels but enter into the lymphatic vessels and accumulate in the LNs (12). Moreover, CNSI with the features of black and strong colored ability makes the LNs more readily identified and makes the procedure easier. In addition, CNSI is characterized by slow metabolism and can be observed *in vivo* after approximately 3-4 months. Multiple studies have reported that CNSI has a respectably high safety profile, and no significant adverse effects were seen while the detection rate of LNs and metastatic LNs was increased (11, 13). Additionally, researchers have found no suggestion of increased complication rates or operating time.

ICG, as a kind of fluorescent dye, can be applied intraoperatively and used to sort LNs in postoperative specimens. When injected into the submucosa of the gastric wall of GC patients, ICG combines with serum albumin in the circulation and can be found in the LNs. Once excited by infrared light (wavelength, 750–810 nm), ICG can emit infrared fluorescence (peak wavelength, approximately 840 nm), so ICG fluorescence imaging can guide intraoperative LN dissection (14, 15). ICG fluorescence imaging can be performed successfully under a laparoscopic or robotic imaging system (16). Compared with other dyes, ICG fluorescence imaging with better tissue penetration has the potential to identify the LNs shaded by hypertrophic adipose tissue. ICG-guided D2 LN dissection has become a novel hot topic explored by an increasing number of gastrointestinal surgeons. In addition, ICG fluorescence imaging increases the number of LNs retrieved and exhibits a good clinical efficacy and safety profile (15–18).

Concerning the LN metastasis rates, there was not much difference between the CNSI, ICG, and control groups. Hence, we concluded that CNSI was more likely to be the best LN tracer at present. However, this is a retrospective single-center study that includes the well-known limitations of such a study design. It is therefore worthwhile to carry out a prospectively designed study to compare the safety and efficacy of CNSI and ICG tracer-guided LN dissection during radical gastrectomy.

The present study therefore aims to compare the safety and efficacy of CNSI and ICG tracer-guided LN dissection during radical gastrectomy using a randomized clinical trial design and to provide a theoretical basis for further investigation.

## 2 Methods and analysis

### 2.1 Study design

**Figure 1:**
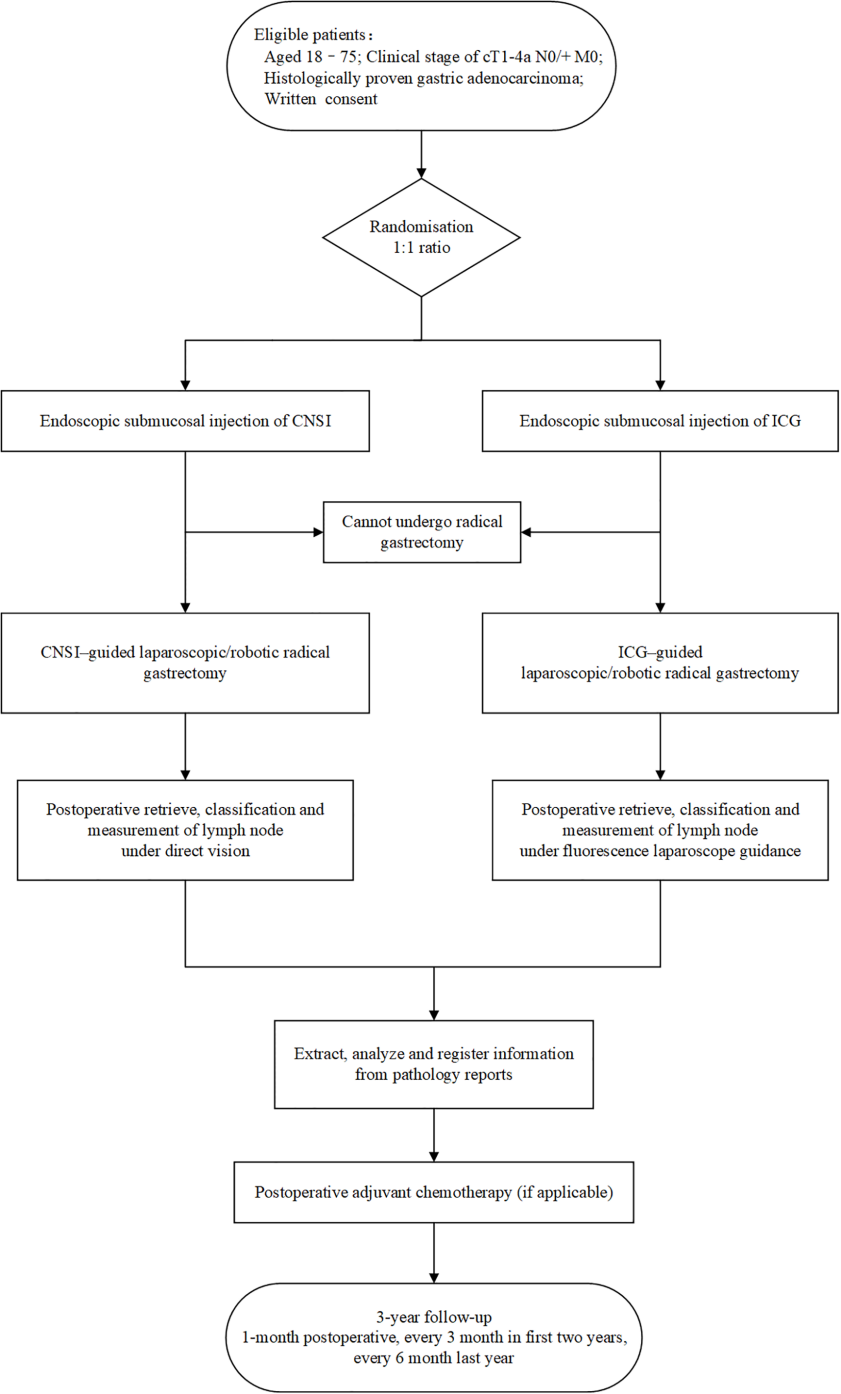
Flow chart.

The current study is a prospective, randomized, open label, single-center, noninferiority clinical trial. The study takes place in the Fourth Hospital of Hebei Medical University. Patient enrollment started on 20 January 2021, and the trial is expected to end on January 20 2025. **Figure 1** summarizes the design of the trial, and each of the trial aspects is described in detail below.

### 2.2 Eligibility criteria

The inclusion criteria are as follows: (1) aged 18–75; (2) histologically proven gastric adenocarcinoma on biopsy (papillary adenocarcinoma, tubular adenocarcinoma, mucous adenocarcinoma, signet-ring cell carcinoma or poorly differentiated adenocarcinoma); (3) proven clinical stage of cT1-4a N0/+ M0 by ultrasound endoscopy, enhanced CT/MRI examination or diagnostic laparoscopy according to the TNM classification of the American Joint Committee on Cancer (AJCC cancer staging manual, eighth edition); (4) without distant metastasis and no invasion of adjacent organs; (5) a preoperative Eastern Cooperative Oncology Group (ECOG) score of 0 or 1; (6) a preoperative American Society of Anesthesiologists (ASA) score of I-III; and (7) signed a written informed consent form.

The exclusion criteria are as follows: (1) pregnant or lactating women; (2) patients with severe mental disorder; (3) a history of upper abdominal surgery (with exception of laparoscopic cholecystectomy); (4) a history of gastrectomies, endoscopic mucosal resection (EMR) or endoscopic submucosal dissection (ESD); (5) enlarged or bulky regional LNs according to preoperative imaging and larger than 3 cm at the long diameter; (6) the tumor invades the duodenum or esophagus; (7) Borrmann type IV GC; (8) history of other malignant tumors within the last 5 years; (9) received previous neoadjuvant radiotherapy or chemotherapy; (10) history of myocardial infarction or unstable angina within the last 6 months; (11) history of cerebrovascular accident within the last 6 months; (12) undergoing sustained systemic corticosteroid treatment within 1 month prior to surgery; (13) emergency surgery due to GC complications (bleeding, perforation, or obstruction); and (14) FEV1 <50% predicted value.

### 2.3 Randomization and blinding

The patients were enrolled by a dedicated surgeon on our team. For eligible patients treated with GC at The Fourth Hospital of Hebei Medical University, thoracoabdominal CT, echocardiography, ultrasound of supraclavicular LNs, sonography of lower extremity veins, electrocardiogram and lung function examination should be performed before the operation. The patients who meet the inclusion criteria and are feasible for radical onco-surgery are then randomized to receive either endoscopic injection of CNSI (CNSI group) or endoscopic injection of ICG (ICG group) during gastroscopy at a 1:1 ratio. Randomization has been achieved using a random number table by the data manager, and the allocation is not concealed. While the blinding of the surgeons or participants is impossible, the pathologists are blinded to the types of surgical approaches.

### 2.4 Interventions

**Figure 2:**
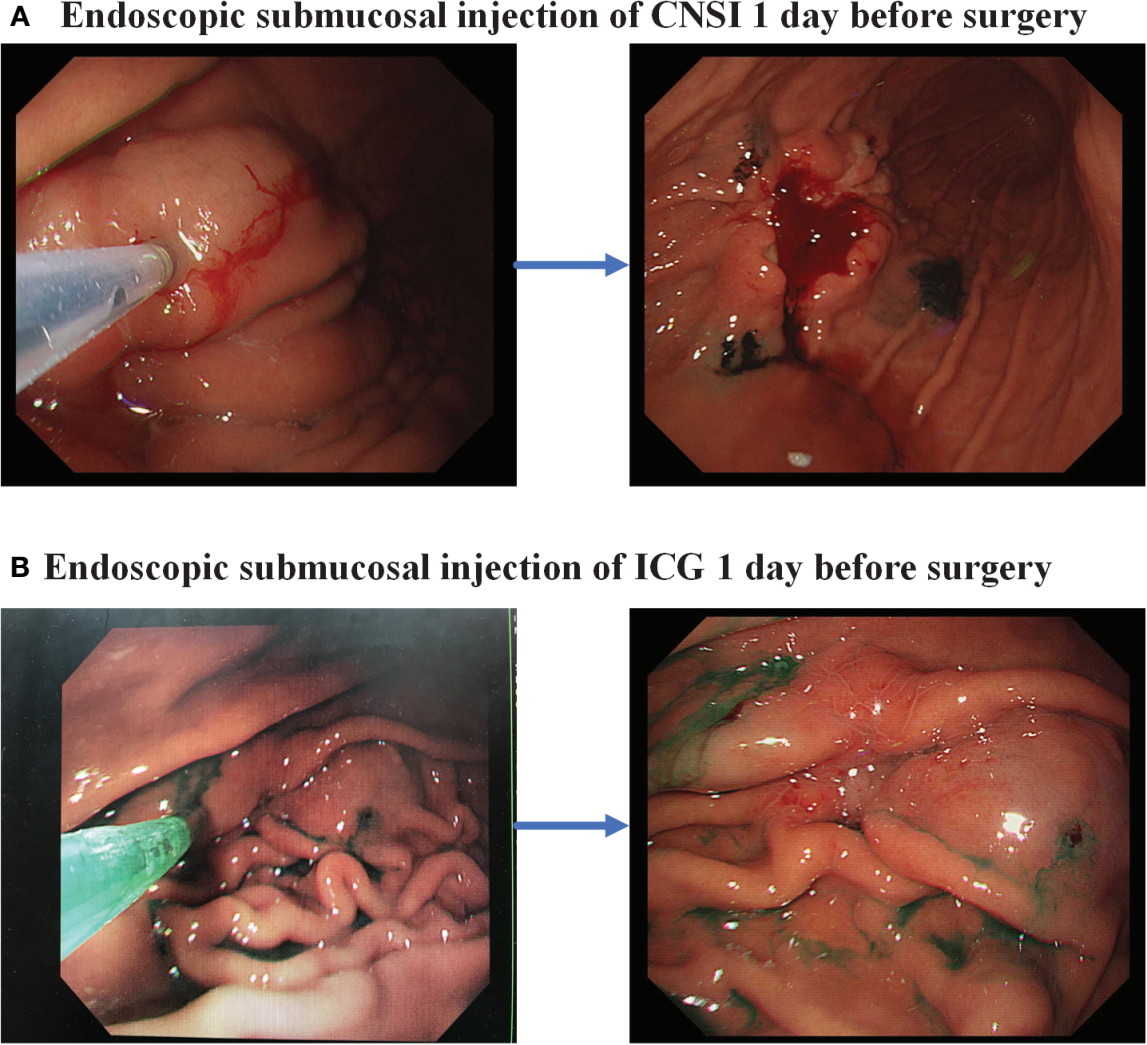
Endoscopic injection of lymph node tracer. **(A)** Carbon nanoparticle suspension injection; **(B)** Indocyanine green.

In the CNSI group, the patients are given injections of CNSI (50 mg/dose) produced by Chongqing Lummy Pharmaceutical Co., Ltd. in the endoscopy division 1 day before surgery. CNSI is injected submucosally at 4 points (proximal side, distal side, and left and right sides) 0.5-1 cm from the tumor edge under endoscopy. The optimized dose for each point is approximately 0.25 ml (**Figure 2A**).

For those who were randomly assigned to the ICG group, ICG (25 mg/dose) produced by Dandong Yichuang Pharmaceutical is marked in the endoscopy division 1 day before surgery and is injected submucosally at 4 points (proximal side, distal side, and left and right sides) 0.5-1 cm from the tumor edge under endoscopy. The optimized dose for each point is approximately 0.5 ml (**Figure 2B**).Both procedures are performed by a designated, experienced endoscopic specialist.

Laparoscopic exploration and detection of free peritoneal cancer cells are necessary in the first step of the surgical procedure to exclude adjacent organ infiltration and peritoneal metastasis. When the patient is positioned in the reverse Trendelenburg position (described as the foot-down and head-up supine position), 800 ml of normal saline is routinely used to wash the area near the carcinomatous foci of GC and is then re-collected as much as possible. It is important to collect at least 300 ml of the flushing fluid from the pelvic cavity. Cytology examination is then immediately performed on the flushing fluid collected. If the peritoneum is free of metastasis and peritoneal lavage cytology is negative, standard laparoscopic or robotic radical gastrectomy with D2 lymphadenectomy is then performed by the designated and experienced team of surgeons according to the Japanese Gastric Cancer Treatment Guidelines 2014. Distal gastrectomy for LN dissection included LN station 1, 3, 4 sb, 4d, 5, 6, 7, 8a, 9, 11p, 12a.

**Figure 3:**
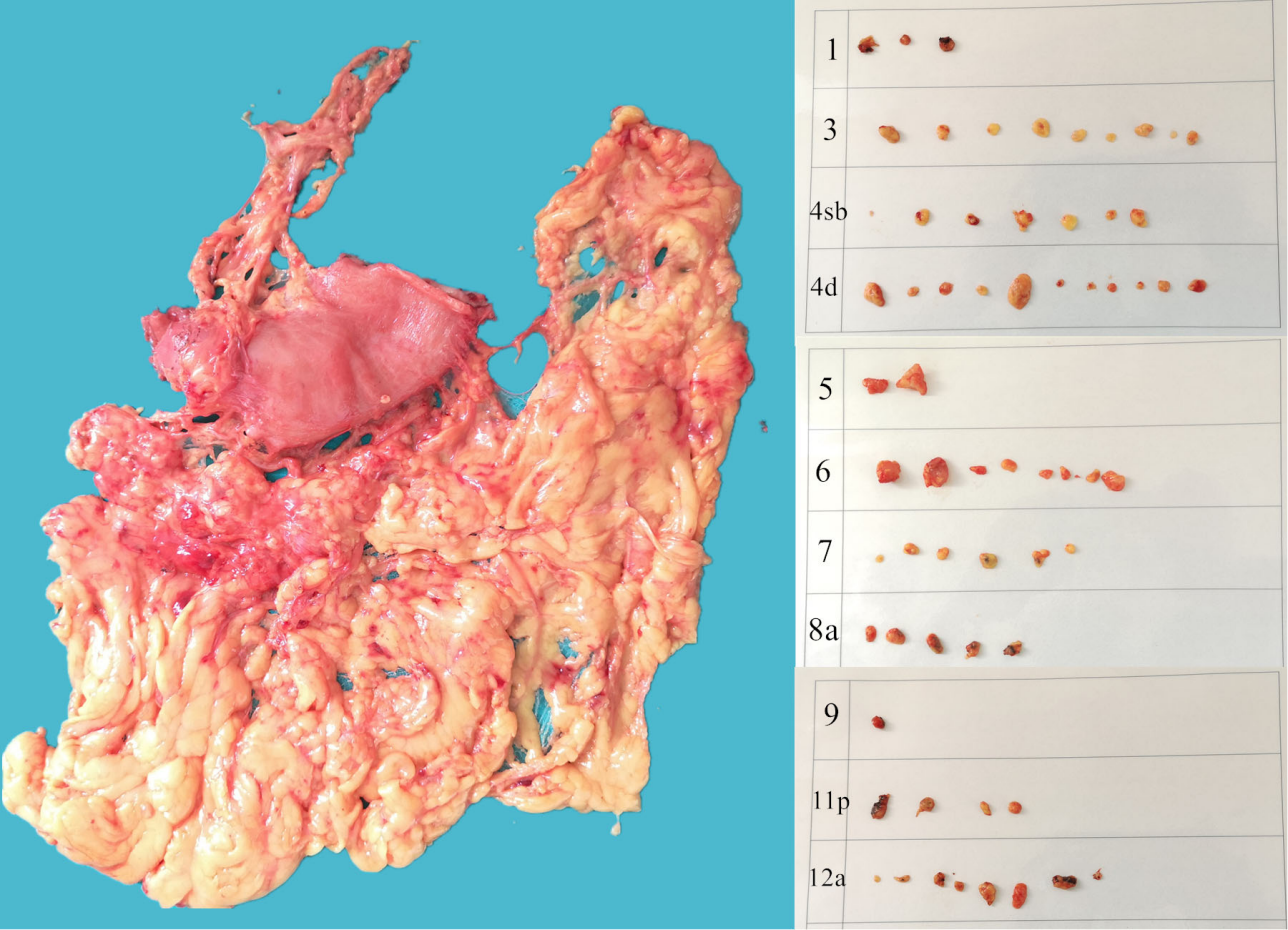
The lymph nodes after dissecting the lymph nodes from specimen.

Before starting to retrieve LNs from the GC patients, the surgeon should determine the positions of each station LN on the specimen in detail and plan the sequence of retrieving the LNs. After washing and flattening the specimen, we began to retain the soft tissues of each station of LNs by cutting off the useless tissues in the specimen (**Figure 3**). Then, after observing the course of the artery encased in fatty tissue and removing the perivascular fat, we dissected the LNs arranged along the vascular lumen. Dissecting the LNs must be completed within 30 minutes after the specimen is detached. The dissected LNs, which are fixed with formalin, were then separated by the different LN stations, the staining status and the LN maximum diameter (>2 or ≤2 mm) and sent to the pathology department for histopathological study. The surgeon appointed to the dissection of the LNs must have a thorough grasp of the knowledge about LNs and abundant experience.

### 2.5 Outcomes

The primary outcome of this study is to determine the number of LNs retrieved. The secondary outcomes are to determine the LN staining (or fluorescence) rate (Number of staining or fluorescence LNs/Total number of retrieved LNs), LN staining (or fluorescence) rate at each station, stained (or fluorescent) LN positive rate(Number of staining or fluorescence positive LNs/Total number of positive LNs), stained (or fluorescent) LN negative rate(Number of staining or fluorescence negative LNs/Total number of negative LNs), nonstained (or nonfluorescent) LN positive rate(Number of nonstaining or nonfluorescence positive LNs/Total number of positive LNs), nonstained (or nonfluorescent) LN negative rate(Number of nonstaining or nonfluorescence negative LNs/Total number of negative LNs), LN metastasis rate(Number of metastasis LNs/Total number of retrieved LNs), number of metastatic LNs, complication and mortality rates within 30 days, 3-year disease-free survival (DFS), 3-year overall survival (OS), recurrence pattern within 3 years, intraoperative blood loss volume, time spent retrieving LNs, and postoperative recovery (exhaust time after surgery, feeding time after surgery, duration of postoperative hospital stay).

Any complications should be recorded and reported. There are mainly two complications related to the procedure: intraoperative complications and postoperative complications. The former includes bleeding, injuring the viscera, lymphatic leakage and so on. The latter should be classified according to the Clavien−Dindo classification system.

### 2.6 Adverse events

Adverse events (AEs) refer to patient injuries caused by medical care (19). Serious adverse events (SAEs) refer to medically related events that result in death, are life-threatening, require hospital admission or prolong hospital stay, or cause persistent or significant disability. Although adverse effects of CNSI and ICG have not been observed in preclinical studies, any forms of adverse events should be systematically recorded.

### 2.7 Sample size

The sample size for this study was calculated based on previous research by our team and computed by PASS version 11. Eligible subjects were randomly assigned (1:1) into the CNSI group and the ICG group. All analyses were two-sided with α=0.05 and β=0.80. the prospective mean number of the LNs retrieved in the CNSI group was 56.93. The prospective mean number of the LNs retrieved in the ICG group was 50.52. The total sample size was 96 (48 per group) after taking into account a 10% dropout rate in each group. The planned recruitment period is 1 year, and the follow-up duration is 3 years.

### 2.8 Data collection and analysis

Designated and trained surgeons collect the data from the pathology reports and operative reports. The focus of the data should contain clear information about the tumor location, tumor size, histopathologic type, tumor differentiation degree, Lauren classification, vascular tumor embolus, nerve invasion, immunohistochemistry results and pathological findings of the LNs at each station. Long-term prognosis data are collected *via* follow-up up until 3 years after the surgical operations. The follow-up should consist of a 3-month interval from 0–2 years and a 6-month interval in the third year. The main follow-up items included routine physical examination, tumor marker detection (CEA, CA199, CA724 and AFP), abdominal CT, and annual electronic gastroscopy. The follow-up included an outpatient follow-up, a telephone follow-up and a short message platform follow-up. Further evaluation and treatment should be performed if relapse occurs.

The purpose of this study is to compare the safety and efficacy of CNSI and ICG during radical gastrectomy. The primary objective is to determine the number of LNs retrieved. Comparability analysis will be performed on the baseline data to determine whether the two groups are comparable. The number of patients, mean, standard deviation, median, maximum, and minimum will be listed. Descriptive data will be presented as the mean ± SD, while categorical data will be presented as the number and percentage (%). The independent samples t test or nonparametric test will be used to analyze continuous data, and the chi-square test or Fisher’s exact test will be used to analyze categorical variables. It is valuable to analyze the influence of CNSI (ICG) staining or other factors on the number of LNs detected according to the statistical analysis. We can also research the effect of CNSI (ICG) staining on the operative time, intraoperative bleeding volume, intraoperative transfusion volume and postoperative complications. Survival analysis will be performed using the Kaplan−Meier method, and the log rank test will be used to test the difference in survival rates between the two groups. SPSS 26.0 statistical software will be used for statistical analysis. P<0.05 will be considered to indicate statistically significant differences.

## 3 Discussion

Despite the serious burden of GC in China, the public awareness of risk factors or warning symptoms and screening is low, and there is a lack of screening programs (20). Therefore, the majority of GC are detected at an advanced stage, and at this moment, LN metastasis usually occurs. It is well known that LN metastasis is one of the most important prognostic factors for GC patients, and D2 LN dissection has gradually become the mainstream treatment to improve patient outcome (21). The mean number of LNs dissected in Western European countries was 29.5, which is slightly higher than that in China (22). This condition might be associated with the insufficient LN dissection intraoperatively and the false idea that postoperative LN dissection should be completed by a pathologist. Currently, an increasing number of studies have begun to focus on LN tracers, and an increasing number of gastrointestinal surgeons have started using carbon nanoparticle suspension injection or indocyanine green.

A number of interesting differences were identified through our analyses of CNSI and ICG. First, taking an appropriate amount of time to dissect LNs as an example, the LNs are stained black using CNSI, while the LNs labeled with ICG have the ability to generate strong fluorescence emission in certain cases. When utilizing CNSI as a tracer of LNs, surgeons can dissect the LNs under direct vision without the assistance of other instruments. However, if surgeons intend to use indocyanine fluorescence imaging to guide the postoperative nodal dissection, the fluorescence imaging mode must be available, and the wear and tear of the instrument and the burden on surgeons and operating room nurses will increase, which may prolong the time needed for dissecting the LNs. Second, compared with CNSI, ICG may have the potential to decrease the risk of anastomotic fistula with the assistance of examining the blood supply of the anastomosis. Third, CNSI extravasation into gastric serosa will contaminate the surgical fields and increase the difficulty of surgically dissecting the gastric tumor, but surgeons can switch between the fluorescence mode and normal mode to avoid the adverse effects caused by ICG extravasation (15).

There are a number of previous reviews on the effectiveness of CNSI and ICG. Chen pointed out that their team analyzed 129 GC patients in the ICG group and 129 control subjects and concluded that ICG tracer-guided LN dissection enabled the retrieval of a higher number of LNs (16). Two retrospective analyses found that fluorescence lymphography has high sensitivity and negative predictive value for the diagnosis of LN metastasis. Fluorescent lymphography-guided lymphadenectomy appears to be a reasonable alternative to conventional systematic lymphadenectomy for gastric cancer (23, 24). Similarly, in accordance with the study of Yan, carbon nanoparticles have the same efficacy and safety as a LN tracer in the clinic (11). Li et al. and our team found that CNSI is a safe material. Surgeons could harvest more LNs in patients with GC. The harvest of an increased number of smaller diameters of LNs may be beneficial. CNSI is associated with facilitating the dissection of all positive LNs, which could improve surgical quality (13, 25). However, very few studies have focused on the comparison of CNSI and ICG. A retrospective study recently conducted by our team suggested that the mean numbers of LNs and micro LNs retrieved in the CNSI group were higher than those in the ICG and control groups, and there were no differences between the ICG and control groups (15).

Whether the results of retrospective analysis can be confirmed by prospective randomized controlled clinical trials still needs to be further studied. Therefore, we are currently conducting a clinical trial to compare CNSI and ICG in detail to choose the optimal and suitable LN tracer for radical gastrectomy.

## Data availability statement

The original contributions presented in the study are included in the article/supplementary material. Further inquiries can be directed to the corresponding author.

## Ethics statement

The studies involving human participants were reviewed and approved by the Ethical Review Committee of Hebei Medical University. The patients/participants provided their written informed consent to participate in this study.

## Author contributions

Conceptualization: QZ and YT. Data curation: YT, YP, PY, HG, YaL, ZeZ, PD, and TZ. Investigation and experiments: YoL, LF, ZhZ, XZ, BT, DW, and QZ. Methodology: YT. Formal analysis: YT, YP, and PY. Supervision: QZ. Writing – original draft: YT and YP. Writing review and editing: YoL, LF, ZhZ, XZ, BT, DW, and QZ. All authors contributed to the article and approved the submitted version.
